# Emerging Medical Treatments for Meningioma in the Molecular Era

**DOI:** 10.3390/biomedicines6030086

**Published:** 2018-08-06

**Authors:** Fares Nigim, Hiroaki Wakimoto, Ekkehard M. Kasper, Linda Ackermans, Yasin Temel

**Affiliations:** 1 Brain Tumor Research Center, Massachusetts General Hospital, Harvard Medical School, Boston, MA 02114, USA; fnigim@mgh.harvard.edu or f.nigim@gmail.com; 2 Department of Neurosurgery, Massachusetts General Hospital, Harvard Medical School, Boston, MA 02114, USA; 3 Department of Neurosurgery, McMaster University, Hamilton, ON 8L8 2X2, Canada; kaspere@hhsc.ca; 4 Department of Neurosurgery and Neuroscience, Maastricht University Medical Center, 6229 HY Maastricht, The Netherlands; linda.ackermans@mumc.nl (L.A.); y.temel@maastrichtuniversity.nl (Y.T.)

**Keywords:** World Health Organization (WHO), Grade I, II, and III Meningiomas, High Grade Meningiomas (HGMs), tumor heterogeneity, genetic subtypes, overall survival (OS), progression free survival (PFS), targeted therapy, clinical trials, oncolytic virus (OV)

## Abstract

Meningiomas are the most common type of primary central nervous system tumors. Approximately, 80% of meningiomas are classified by the World Health Organization (WHO) as grade I, and 20% of these tumors are grade II and III, considered high-grade meningiomas (HGMs). Clinical control of HGMs, as well as meningiomas that relapse after surgery, and radiation therapy is difficult, and novel therapeutic approaches are necessary. However, traditional chemotherapies, interferons, hormonal therapies, and other targeted therapies have so far failed to provide clinical benefit. During the last several years, next generation sequencing has dissected the genetic heterogeneity of meningioma and enriched our knowledge about distinct oncogenic pathways driving different subtypes of meningiomas, opening up a door to new personalized targeted therapies. Molecular classification of meningioma allows a new design of clinical trials that assign patients to corresponding targeted agents based on the tumor genetic subtypes. In this review, we will shed light on emerging medical treatments of meningiomas with a particular focus on the new targets identified with genomic sequencing that have led to clinical trials testing novel compounds. Moreover, we present recent development of patient-derived preclinical models that provide platforms for assessing targeted therapies as well as strategies with novel mechanism of action such as oncolytic viruses.

## 1. Introduction

Meningiomas are tumors that arise from the membranes surrounding the brain and spinal cord, and they are the most common intracranial tumors. Approximately 80% of meningiomas are benign tumors, and a subset (15–20%) are atypical World Health Organization (WHO) grade-II and anaplastic (WHO grade III), also called high-grade meningiomas (HGMs) [1]. HGMs exhibit an aggressive behavior characterized by high recurrence rates and resistance to standard treatments. Nevertheless, a recent study has proposed a new classification system based on DNA methylation profiling to stratify meningiomas and found it to correlate better with progression-free survival and clinical outcome than WHO-grading [2]. Current treatments for HGMs include surgical resection, followed by radiotherapy [1]. The role of chemotherapy is not clear, and it is currently rarely used in clinical practice due to lack of evidence. Clinical trials testing interferon and hormonal agents have shown very minimal effects [3]. One of the challenges in developing effective medical therapies for meningioma is the lack of clinically relevant preclinical animal models that reproduce the tumor heterogeneity enabling predictive studies on meningioma. Heterogeneity is one of the main causes of failure of novel treatments; in most trials patients are enrolled based on their WHO grade and resistance to the standard of care as opposed to genetic driver mutations. Nevertheless, in the last 5 years, next generation sequencing has dissected the molecular heterogeneity and enriched our knowledge about the genetic drivers that could subdivide meningiomas based on molecular backgrounds. This has opened opportunities for new targets and novel treatments to test in the clinics based on the identified genetic mutations. We speculate that trials based on genetic sequencing for each patient will have much higher success rates than previous trials that included heterogeneous patient populations. In this review, we overview published studies on meningioma treatments. We highlight emerging medical approaches targeting pathways identified with next generation sequencing for meningiomas that hold great promise to change the current meningioma treatment paradigm. Moreover, we present the most recent preclinical platforms including patient-derived animal models developed to study meningioma biology and test treatments with novel mechanism of action such as oncolytic virus.

## 2. Genetic Background

Germline mutations found in familial syndromes lighted us about the common genetic alterations in meningioma. One of the most thoroughly described genes is *NF2*, which encodes the tumor suppressor protein Merlin. The *NF2* gene on chromosome 22 is mutated in Neurofibromatosis type II syndrome, and nearly 70% of these patients develop multiple meningiomas of different WHO grades, with grade I being the most common, along with other central nervous system tumors [4]. Furthermore, up to 60% of patients with sporadic meningioma have allelic inactivation or loss of *NF2* [5,6]. *NF2* is considered an initial driver of meningioma as it is mutated in both low- and high-grade meningioma [7], and mice with *NF2*-knockout develop spontaneous meningioma [8].

The majority of meningiomas, are sporadic and harbor different chromosomal aberrations and somatic mutations relevant to their biology (Table 1). Further studies to clarify how each one of these genetic lesions contributes to meningiomagenesis will advance our biological insights and potentially develop novel medical treatments. Monosomy of chromosome 22 is observed in 40–70% of meningioma cases, supporting a role of *NF2*. Although deletions of chromosome 22 affect prevalently the *NF2* gene [5,6], there are a variety of extents in chromosome 22 loss that do not affect *NF2*. This suggests the presence of adjacent genes on chromosome 22q that play a role in meningioma tumorigenesis. These candidates include *SMARCB1*, checkpoint kinase 2 (*CHEK2*), and clarthin heavy chain polypeptide gene (*CLH-22*/*CTCL1*) [5,9].

HGMs have higher propensity to copy number alterations [10]. HGMs frequently harbor loss of chromosomes 1p, 6q, 10, 14q, and 18q, as well as gain of chromosomes 1q, 9q, 12q, 15q, and 20q [10,11,12]. The losses of chromosomes 1p and 14q are the second most frequent cytogenetic alterations observed in meningioma after chromosome 22 loss, and they affect 50% of grade II and almost all grade III meningiomas [12]. Changes in chromosome 1p involve multiple genes; some are being investigated as potential meningioma driver genes including *TP73*, *CDKN2C*, *RAD54*, *EPB41*, *GADD45A*, and *ALPL* [13,14,15,16]. On chromosome 14q, tumor suppressor genes *NDRG* and *MEG3* are found to be inactivated in malignant meningiomas [17,18]. Frequent deletions or inactivating mutations affecting cell cycle genes on chromosome 9p in HGMs are found to predict poor outcome and shorter survival [19]. These genes on chromosome 9p21 include *CDKN2A* (encoding INK4a/p16) and *CDKN2B* (encoding INK4b/p18), inhibiting the CDK4 and CDK6 cyclin-dependent kinases, respectively [19]. ARF/p14 promotes TP53 activity through repression of the murine double minute 2 (MDM2) protein, which is a TP53 inhibitor [20].

Next generation DNA sequencing has vastly enriched our knowledge about the recurrent driver mutations that occur in meningioma and changed the concept of the genetic status of meningioma. Besides *NF2*, 4 genes, *SMO*, *TRAF7*, *AKT*, and *KLF4* have been identified from a series of whole genome and exome sequencing efforts conducted on clinical meningioma specimens; these mutations are present in 40% of sporadic meningiomas and are mutually exclusive with chromosome 22 mutations including *NF2* [5,21,22]. Mutations in the hedgehog pathway signaling member smoothened (*SMO*, *7q32.1*) have been observed in 5.5% grade I meningioma and they are either L412F or W535L [5,21]. A recent study conducted by Boetto J et al. reported 22 tumors of 79 olfactory groove meningiomas analyzed to be carrying SMO L412F (21 tumors) or W535L mutation (one tumor) [23]. Another group reported similar finding where olfactory groove meningiomas tend to have higher SMO mutation rates than meningiomas at other locations [24].

In total, 12–25% of meningiomas grade I were found to have mutations affecting *TRAF7*, encoding a proapoptotic N-terminal RING and zinc finger domain protein with E3 ubiquitin ligase TNF-receptor associated factor 7 (TRAF7), located on chromosome 16p13 [5,22]. TRAF7 potentiates MEKK3-mediated signaling and regulates activation of NF-κB signaling [25]. In breast cancer, suppression of *TRAF7* gene expression was associated with TP53 accumulation, which is reported to be due to the absence of *TRAF7*-mediated TP53 ubiquitination [26]. In meningioma, *TRAF7* mutations can co-occur K409Q mutation in KLF4, a transcription factor known for its role in inducing pluripotency, and are mutually exclusive with *NF2* mutations, chromosome 22 loss, and *SMO* mutations and was less common in HGMs [5,6,21,22]. Meningioma with *TRAF7* mutations can harbor mutations in *KLF4* or *AKT1* in 40% and 33% of cases, respectively [5,22,27].

*AKT1* is the proto-oncogene murine thymoma viral oncogene homolog 1, located on chromosome 14q32. *AKT1* p.E17K results in constitutive activation of *AKT1* that promotes proliferation and tumor growth [28,29]. *AKT1* mutation appears to be mutually exclusive with *NF2*, *SMO* and *KLF4* mutations [5]. The fourth affected gene is *KLF4*, the pluripotency transcription factor Kruppel-like factor 4 (KLF4), located on chromosome 9q3. *KLF4* is a member of a family of DNA-binding transcriptional regulators involving proliferation, differentiation, migration, inflammation, and pluripotency [30,31]. Nevertheless, *KLF4* is reported to be a tumor suppressor in some cancers such as pancreatic ductal cancer, lung cancer and colorectal cancer [32,33]. *KLF4* mutation is always c.1225A > C (p.K409Q) and it is specific for meningiomas; 15.7% of grade I meningioma and 9% of all meningiomas combined harbor this mutation [5,22]. All *KLF4* mutations co-occur with *TRAF7* mutations and are mutually exclusive with *NF2* and *AKT1* mutations [5].

*TERT* gene (*5p15.33*) promoter mutations are often identified in meningioma and are associated with recurrence and malignant progression [34,35]. The hotspot mutations are C228T and C250T, [34], which are present in various tumors including melanoma, urothelial carcinoma, hepatocellular carcinoma, glioblastoma, and oligodendrogliomas [36,37,38,39,40,41]. Both C228T and C250T lead to transcriptional activation of TERT by 2 to 4 folds [36,37]. These mutations are found in both *NF2* and non-*NF2* meningiomas [34]. Patients with meningioma with *TERT* mutations have shorter survival time and time to progression after treatment, suggesting the role of *TERT* promoter mutation in tumor evolution and progression [35]. Two meningioma cell lines, IOMM-Lee and CH-157 MN, harbor the C228T hotspot *TERT* promoter mutation and could be useful to develop and test *TERT* inhibitors given their current lack of clinical testing [42]. Development of *TERT* inhibitors could have a large indication not only in meningioma but also in other cancers that harbor *TERT* promoter mutation by reversing the phenotype cancer acquires with hyperactive *TERT*.

Other genetic alterations found in meningioma include *POLR2A*, *PIK3CA*, *SMARCE1* and *BAP1*, and their clinical relevance is summarized in Table 1 [21,22,43,44].

## 3. Clinical and Histological Features in Connection to Genotypes

There is no correlation between genotypes and patient age, median age reported was between 50–60 years old, across all genetic mutations [45]. There is strong association between genotypes and tumor location (Table 1). Over 60% of *NF2* inactivated meningioma is reported to be located in the calvarium, including convexity of the skull, parasagittal region, and falx cerebri. Less frequently *NF2* meningiomas are in the lateral posterior fossa and lateral middle fossa. Half of meningiomas that harbor *SMO* mutation are located in the anterior fossa and the remaining is located usually in the calvarium and median middle fossa similar to *TRAF*/*AKT1* mutant meningiomas. Approximately 70% of *TRAF7*/*AKT1* meningiomas are in the anterior fossa or median middle fossa, and 20–30% are located in the anterior convexity [5,6,10,22,27,46].

Different histological types of meningioma correlate with genetic aberrations. Fibrous meningiomas are more common in *NF2* type meningiomas than with other mutations. Meningothelial and transitional meningiomas are more frequently seen in *SMO*, *TRAF7*/*AKT1*, and *TRAF7*/*KLF4* mutant tumors. Notably, *TRAF7*/*KLF4* mutant meningiomas are the secretory subtype. *NF2* mutant meningiomas are in all grades including WHO grade II and III whereas *SMO*, *TRAF7*, *TRAF7*/*AKT1* and *KLF4* meningiomas tend to be WHO grade I [5,6,10,22,27,46].

## 4. Medical Treatment for Meningioma

### 4.1. Chemotherapy

Overall chemotherapy has been found to be poorly effective in meningioma, as an adjuvant treatment after surgery and radiotherapy. Numerous clinical trials and case series have shown that chemotherapy has minimal role and does not improve patients’ outcomes [47,48,49,50,51,52,53,54]. Hydroxyurea, a ribonucleotide reductase inhibitor, was found to arrest meningioma cell cycle in the S-phase and induce apoptosis. A preliminary report showed that 20 mg/kg/day of hydroxyurea was able to prevent recurrence of malignant meningioma for 24 months in a patient who had complete resection [55,56]. Several small phase II studies using hydroxyurea have shown response rates less than 5%, with 50% of patients achieving stable disease, and the median progression-free survival (PFS) ranging from 44–176 weeks [57,58,59,60,61,62].

Hydroxyurea and imatinib were used for recurrent refractory meningiomas, and while the treatment was well tolerated, the combination treatment did not affect survival [63]. Another group led phase II studies combining hydroxyurea and calcium channel antagonist verapamil for recurrent refractory meningioma [64]. The trial concluded in 2015, but as of now, no results are available. Chamberlein et al, [51] reported the results of a small series of malignant meningioma patients treated with 3–6 cycles of cyclophosphamide, doxorubicin, and vincristine. Treatment was associated with high toxicity and very modest response to treatment: median time to tumor progression of 4.6 years (range 2.2–7.1 years) and median survival of 5.3 years (range 2.6–7.6 years) [51]. The alkylating agent temozolomide was found to have no effect on meningioma, and this could be due to intact activity of the DNA repair enzyme O6-methylguanine DNA methyltransferase (MGMT) in meningioma [65,66]. Irinotecan was found to have an anti-meningioma effect in in vitro and in vivo animal studies but failed to show benefits in a phase II clinical trial [67].

### 4.2. Targeted Therapy

Our understanding of the growth factors and their receptors, and the signal transduction pathways that are critical to meningioma growth is still limited [47,68,69,70,71]. Nevertheless, the importance of deregulated cell signaling pathways as a driver of neoplastic transformation is increasingly apparent in meningioma. Studies in meningioma cells have identified aberrant expression of critical signaling molecules [53,72]. The latter suggests that identifying molecular targets driving tumor cell growth, proliferation and angiogenesis may prove valuable in therapy and some are being tested in clinical trials (Table 2).

In 2013, two groundbreaking studies employing next generation sequencing have reported somatic genetic driver mutations that stratify meningioma patients and identify novel therapeutic targets. Both studies showed the presence of targetable somatic mutations in *SMO* (a key component of the Hedgehog pathway) and *AKT1*^E17K^ (a member of the PI3K/AKT/mTOR pathway) activating the respective oncogenic signaling in a subset of *NF2* wild type, but not in *NF2* mutant, meningioma [5,6].

The Hedgehog (Hh) signaling pathway is crucial for embryogenesis and cells proliferation and was found to be significantly activated in some meningiomas [5,73]. Signaling is initiated by the binding of the secreted morphogen, Hh, to its receptor, patched 1 (PTCH1). In the unbound state, PTCH1 inhibits Smoothened (SMO), a G-protein coupled phosphoprotein receptor, by preventing its localization to the cell surface. In the presence of the Hh ligand, the Hh-PTCH1 complex is internalized and the repression of PTCH1 on SMO is relieved. Surface localization of SMO is thought to initiate a signaling cascade, leading to the activation of the glioma-associated (Gli) family of zinc finger transcription factors, which upregulate genes contributing to proliferation, survival, and angiogenesis. Mutations in genes in the Hh pathway and Hh overexpression cause abnormal signaling activation [74,75]. Mutation in *PTCH1* and *SMO* has been identified in meningioma, medulloblastoma, and basal cell carcinoma, resulting in pathway activation [5,76,77] In basal cell carcinoma that harbors mutations in either *PTCH* or *SMO*, locally advanced and metastatic lesions have been very effectively inhibited by a small molecule SMO inhibitor vismodegib that received FDA approval in 2012 [78]. Therefore, antagonism of excessive Hh signaling could provide a pathway to a specific mechanism-based anticancer therapy in meningioma, preventing tumor growth and causing tumor regression without toxic effects in normal tissue [79].

High expression in AKT pathway was reported in skull base meningiomas [80]. AKT is a serine/threonine protein kinase that has 3 isoforms (AKT1, AKT2, and AKT3) and regulates several cellular processes, including survival, proliferation, tissue invasion, and metabolism. AKT has been reported to be crucial in mediating tumor proliferation, survival, and resistance to chemotherapy and targeted agents. Approximately 8% of meningiomas have *AKT1*^E17K^ mutation, which is almost always c.49G > A (p.E17K) and was identified in multiple cancers such as colorectal, breast, bladder, lung, ovarian and endometrial carcinoma [5,22,29,81,82,83,84,85]. This mutation alters the electrostatic interactions, activating AKT1 in a PI3K-independent manner; it transforms rodent cells in vitro and induces spontaneous leukemia in mice [29]. Recently, a new inhibitor of *AKT1*^E17K^ called AZD5363 was tested and found to be efficacious and safe in patients with advanced solid cancers such as breast cancer. [86].

*FAK* gene amplification and protein overexpression have been shown to be present in meningioma, making FAK an attractive therapeutic target [5]. Focal Adhesion Kinase (FAK) is a non-receptor cytoplasmic protein tyrosine kinase that integrates signals from integrins and growth factor receptors to regulate cell proliferation, survival, migration, invasion, and cancer stem cell (CSC) renewal [87,88,89]. *FAK* gene amplification and protein overexpression have been shown to be present in a variety of cancers, making FAK a potential therapeutic target in other cancers as well [87,90]. The connection between the *NF2* merlin protein and FAK pathway in cancers was first reported by Poulikakos et al. [91]. The group reported that merlin negatively regulates FAK and *NF2*-null malignant mesothelioma has overexpression of phosphorylated FAK; whereas overexpression of merlin in *NF2* mutant cells attenuated FAK phosphorylation at the critical phosphorylation site Tyr397 and disrupted the interaction of FAK with its binding partners Src and p85 [91]. The inhibitory effect of merlin on FAK activity could be both direct through the formation of a complex with FAK and NHERF, [92], or indirect by inhibiting Rac/PAK signaling, which is known to cross-talk with FAK pathway [89,93,94]. FAK inhibitor has been reported to be efficacious against *NF2* mutant ovarian cancer and mesothelioma in vitro and in vivo [95,96]. Following these preclinical findings, [95,96], multiple trials are being conducted using FAK inhibitor for mesotheliomas and other advanced solid cancers that harbor *NF2* mutation and lack merlin (ClinicalTrials.gov Identifier NCT01778803, NCT01870609, NCT02004028, NCT01938443, NCT02546531). The clinical data of these trials will determine whether FAK inhibitors affect clinical outcomes in merlin-deficient cancers.

Identification of meningioma genetic subtypes and respective aberrant signaling has provided a scientific rationale for a new clinical trial design that simultaneously investigates different targeted agents according to tumor genotypes. A multi-institutional clinical trial started in August 2015 with 3 arms; patients with tumors that harbor mutation in *SMO*, *AKT1* or *NF2*, receive SMO inhibitor (vismodegib), AKT1 inhibitor (afuresertib) or oral FAK inhibitor (GSK2256098), respectively (clinical trial gov Identifier NCT02523014). To our knowledge, those are the first meningioma trials that target aberrant pathways caused by specific gene mutations identified with the next generation sequencing. Those genetic biomarkers should help us in both patient stratification and follow up during and after the treatment. Clinical data that those trials generate could not only identify promising targeted agents but also provide support for genetic testing in the clinical practice.

mTOR is an evolutionary conserved serine/threonine kinase and is highly active in meningioma tumors, especially skull base meningiomas [97]. mTOR regulates cell growth, proliferation, and survival mainly through 2 distinct functional complexes, mTORC1 and mTORC2, which signal to specific downstream pathways [98,99]. mTORC1 phosphorylates p70S6K and 4EBP1 whereas mTORC2 phosphorylates AKT, PKC-α, and SGK1 [100]. mTORC1 is known to be constitutively activated in *NF2*-associated meningioma, and drugs such as rapamycin were shown to block mTORC1 activation and inhibit *NF2*-meningioma and schwannoma growth in vitro and in vivo [101,102,103,104]. These results have led to clinical trials with the mTORC1 inhibitor everolimus (RAD001), a rapamycin analog, for patients with *NF2* vestibular schwannoma. Everolimus as a single agent has been reported to yield no radiographic response or clinical improvement in patients with *NF2* vestibular schwannomas [105,106,107]. Currently, a phase 0 clinical trial (NCT01880749) is including patients with vestibular schwannomas and meningiomas with a primary end-point to study the pharmacodynamics and kinetics of everolimus. The results of a phase II clinical trial (NCT00972335) combining bevacizumab (anti-VEGF) and everolimus in recurrent progressive meningioma have been reported [108]. The study included 17 patients with different grades of progressive and refractory meningiomas; patients received both drugs for 28 days after surgery and radiotherapy. Overall median progression free survival (PFS) was 22 months, and the median duration of disease stabilization was 10 months. The treatments were well tolerated overall, but 4 patients had to discontinue the treatment due to grade 1 and 2 toxicity [108]. The combination of everolimus and octreotide (somatostatin agonist) was found to significantly inhibit meningioma cell proliferation in vitro. [109] Based on this promising preclinical data, a phase II clinic trial has started in 2015 (NCT02333565) to test the combination of everolimus and octreotide in patients with recurrent meningioma. Both trials - NCT00972335 and NCT02333565 - included both *NF2* wild type and mutated meningiomas, and no results are available as of now.

Aberrant activation of mTORC1 in *NF2*-null meningioma and human arachnoidal cells was found, and showed rapamycin inhibition of cellular proliferation was driven by *NF2* loss. In addition, dual inhibition of both mTORC1 and mTORC2 with AZD2014 was more effective than rapamycin, which inhibits only mTORC1, in inhibiting human primary meningioma cells with *NF2* deficiency [100]. Based on these promising preclinical findings, a phase II clinical trial of AZD2014 was initiated (NCT02831257). This single arm study is currently enrolling patients with neurofibromatosis 2 with progressive or symptomatic meningioma. The primary endpoint of the study is radiographic response and the secondary endpoint is median progression free survival and overall survival (NCT02831257).

### 4.3. Epidermal Growth Factor Receptor (EGFR)

EGFR is expressed in more than 60% of meningioma [110]. EGF and TGF-α activate EGFR pathway and appear to induce meningioma cell growth in culture [68,111]. Thus, activation of EGFR may contribute to proliferation of human meningioma. Multiple groups have reported aggressive growth of meningiomas that have increased TGF-α activity [68,112]. Two single-arm clinical trials were conducted by the North American Brain Tumor Consortium (NABTC) testing two EGFR inhibitors in recurrent meningioma. In NABTC 00–01, 16 patients with recurrent or progressive meningioma were treated with 500 mg/day of gefitinib, an EGFR inhibitor. In NABTC 01–03, nine patients received 150 mg/day of erlotinib, another EGFR inhibitor. These trials included 8 patients with grade I (benign) tumors, nine with grade II (atypical), and eight with grade III (malignant). In both trials, the drugs were well tolerated without toxicities [113]. No objective imaging responses were found; eight patients had a stable disease that was considered due to the treatment. But neither drug appeared to have significant effects against recurrent meningioma [113].

The blood brain barrier (BBB) is not considered a problem in drug delivery to meningioma as tumors are typically localized outside the central nervous system. In a phase I clinical trial, a murine monoclonal antibody against EGFR made in Cuba was tested in 9 patients with meningioma or glioma, but yielded no radiographic responses. However, efficacy data is difficult to interpret and generalize due to the small cohort size. Larger cohorts are required to draw any conclusion regarding the role of this therapeutic in meningioma therapy [114].

### 4.4. Platelet-Derived Growth Factor Receptor (PDGFR)

Meningiomas express platelet-derived growth factor (PDGF) and its receptors [115]. Higher expression of PDGF was reported in atypical and malignant meningiomas than in benign meningiomas [116]. Preclinical studies showed that the supplementation of PDGF-BB increased meningioma cell proliferation while anti-PDGF-BB antibodies had the opposite effect [117]. A phase II clinical trial in patients with recurrent meningioma used Imatinib mesylate, a potent inhibitor of PDGF receptor α and β [118]. The study enrolled 23 patients (13 benign, five atypical and five malignant meningiomas); patients initially received 600 mg/day of Imatinib in the first cycle and 800 mg/day in the second cycle. Although the drug was well tolerated, and no toxicity reported. The agent had minimal activity and no radiographic responses were found. For benign meningioma, median PFS was 3 months (range 1.1–34 months) and PFS6 was 45% across all patients. For atypical and malignant meningiomas, median PFS was two months (range 0.7–3.7 months) and PFS6 was 0% [118]. Imatinib was used in combination with hydroxyurea but failed to show any benefit [63].

### 4.5. Anti-Angiogenesis

Inhibition of cancer-associated blood vessels has become an important approach in cancer treatment [119,120]. Meningiomas are known to have high vascularization and therefore interfering with the tumor nutrition by diminishing its vascular supply could be therapeutically beneficial [121]. An early study reported by Yazaki et al. showed how anti-angiogenic drug fumigillin analogue (TNP-470) inhibited the growth of benign and malignant meningioma in xenografts animal models [122]. One of the most important molecules that drive tumor vascularization is vascular endothelial growth factor (VEGF), and anti-VEGF drugs are approved and used in clinics for different cancers [119,120]. Meningiomas do express VEGF and VEGF-R, and this expression directly correlates with tumor grade as atypical meningiomas and malignant meningiomas express 2- and 10-fold higher levels of VEGF, respectively, than benign meningiomas [123]. Multiple meningioma clinical trials tested the anti-VEGF antibody bevacizumab [124,125,126,127,128,129,130]. Partial regression of an *NF2*-deficient meningioma was reported in a patient who received bevacizumab intravenously every two weeks for 15 months after seven non-curative surgeries [127]. Another one-case study reported partial radiological regression of anaplastic meningioma in a patient who received bevacizumab [126].

A retrospective study was conducted on 15 patients with atypical or malignant meningioma who received bevacizumab [130]. All patients tolerated the treatment well; median PFS was 26 weeks, and PFS6 rate was 43.8%. The group reported a decrease in the tumor blood volume measured with MR perfusion studies in one patient and two patients had decreased enhancement in MRI that did not meet the criteria for response based on the Response Assessment in Neuro-Oncology (RANO) criteria [130,131]. Another retrospective study used bevacizumab in 15 patients with recurrent atypical and malignant meningioma and reported that PFS6 was 86% [129]. Bevacizumab was in general well tolerated but one case had central nervous system (CNS) hemorrhage and another case had intestinal perforation [129]. A regression of a grade I meningioma was reported in a patient who had triple negative breast cancer who received bevacizumab and paclitaxel. One year after regression, her MRI continued to show stable regression of the meningioma [128].

Dual inhibitors of VEGFR and PDGFR (e.g., Sunitinib and Vatalanib) have been developed to inhibit neoplasms, in part, through interfering with tumor vascularization. A prospective randomized phase II clinical trial of sunitinib was conducted on 36 patients with atypical and malignant meningioma [125]. Sixty percent of patients experienced grade 3 toxicities, 32% of patients required dose reduction and 22% of patients were removed from the study. Toxicities included CNS hemorrhage, GI symptoms and anorexia [125]. Nevertheless, PFS6 was 42% and it met the primary endpoint. The authors suggested a follow up study to further investigate sunitinib’s efficacy in a larger patient population.

A phase II clinical trial of vatalanib (PTK787), a VEGFR and PDGFR inhibitor, was conducted enrolling 25 patients with benign, atypical and malignant meningioma [132]. On average, four cycles of PTK787 were administered in each patient [132]. PTK787 was safe, and minor toxicities including fatigue, hypertension and elevated transaminases were reported. Grade II patients had PFS6 of 64.3%, median PFS of 6.5 months and OS of 26 months; grade III patients had PFS6 of 37.5%, median PFS of 3.6 months and OS 23 months [132]. Given the promising results of this study, larger prospective phase III randomized clinical trials should be performed to draw conclusions about the role of this treatment in the context of meningiomas from different grades.

### 4.6. Hormonal Therapy

Meningiomas are more common in women with an increase in incidence after puberty and during their reproductive years. Moreover, a large population based case-control study found a direct correlation between the number of pregnancies leading to birth and meningioma risk in women less that 50 years old [121,133,134]. Higher incidence in meningioma was reported among breast cancer patients [135]. Although no evidenced-based studies demonstrated direct correlation between meningioma tumor growth and the reproductive hormone levels, the association between meningioma and reproductive hormones has been found in case reports and retrospective studies that suffer the limitation of patient number and confounding variables [136,137].

Estrogen receptors (ER) are expressed in 10% of meningioma while progesterone receptor (PR) is expressed in higher percentages of meningiomas [121,138,139,140] HGMs tend to express more estrogen receptors whereas benign meningiomas express progesterone receptor [141]. Estrogen receptor inhibitors and anti-estrogen agents have not shown strong effect in meningiomas [142,143]. In the first case study published in 1985 [143], six patients with inoperable recurrent meningioma received tamoxifen, an anti-estrogenic agent, for a period of 8–12 months. The two-year study results did not indicate any favorable response to tamoxifen, with only one patient showing radiographical partial response [143]. A larger phase II clinical study published in 1993 [142] included 19 patients with refractory meningioma who received tamoxifen. One patient had an MRI-documented response while two had minor responses; six patients had stable disease for over 31 months while 10 patients progressed without any response. Twenty-two percent of patients reported subjective improvement, and there was no significant objective improvement defined as a radiographic response in any of the cases [142]. At present, there is no recommendation for the use of anti-estrogenic agents for meningioma due to lack of evidence of efficacy. Prospective larger studies are required to determine the role of estrogen inhibitors in ERs-expressing meningioma.

High-level expression of PR in meningioma has drawn a lot of attention as a potential target in meningioma treatment [141]. The first study, published in 1991 [144], used PR inhibitor mifepristone (RU486) in 14 patients with unresectable meningioma receiving mifepristone for 2 to 31 months [144]. Five patients showed signs of objective response, defined as a reduction of the tumor measurement on neuroimaging or improved visual field examination. Three patients experienced subjective improvement defined as relief of headache or improved extraocular muscle function. The drug was safe and no high-grade toxicities reported in any patient [144]. Another study included 10 patients with recurrent and unresectable meningiomas that were treated with mifepristone [145]. The study reported decreases in tumor size in three patients and stable disease in other three, with no toxicities [145], although promising, both studies were limited with the small sample size. Neither study reported the WHO grades of patients who received the treatment.

A large prospective randomized 2-arm multicenter study that enrolled 180 patients did not demonstrate the anti-tumor effect of mifepristone in recurrent meningioma. Patients were randomized to either mifepristone or placebo [146]. The median PFS was 10 months in the mifepristone group and 12 months in the placebo group, with overall median survival of 31 months [146]. A phase II clinical trial of 28 patients with unresectable meningioma showed reduction of less than 10% of the tumor area without clinical improvement in eight patients who received mifepristone [147]. So far there is no evidence that supports the use of PR inhibitors in meningioma. Intratumoral heterogeneity of PR expression should be considered for future clinical trials.

Approximately 90% of meningiomas express somatostatin receptors. [148] The addition of somatostatin inhibits meningioma growth in vitro in some studies whereas others have shown the opposite effect [148,149]. A pilot study that included 16 patients with different grades of recurrent meningioma has shown some effect after treating patients with Sandostatin LAR, a somatostatin analogue [150]. Thirty one percent of patients had partial response, 31% had stable disease and 38% had progressive disease. PFS6 was 44% across all patients and the drug was well tolerated [150]. Another study published in 2011 used a novel somatostatin analogue called Pasireotide (SOM230) that binds to and blocks almost all somatostatin receptors [151]. The study included 34 patients with recurrent and progressive meningioma divided in two cohorts, cohort A and cohort B. There was no evidence of radiographic antitumor response to Pasireotide; 67% of patients in cohort A and 81% in cohort B achieved stable disease. PFS6 in cohort A was 17% and PFS 15 weeks, while in cohort B PFS6 was 50% and median PFS was 26 weeks. Treatment was very well tolerated and no toxicities were reported [151]. A more recent phase II study conducted at Mayo Clinic tested octreotide, a somatostatin agonist, in 11 patients with recurrent and progressive meningioma [152]. No anti-tumor radiographic effects were found, and all patients experienced disease progression with median time to progression being 17 weeks and median survival 2.7 years [152]. This study does not show promising results to support a role of somatostatin in meningiomas. Nevertheless, larger randomized studies should be performed to make solid conclusion [144,145]. A recent study reported an antitumor effect of octreotide on a progressive meningioma grade II from grade I after surgery and radiotherapy which stayed in remission for over three years while being treated with octreotide [153].

### 4.7. Interferons

Recombinant interferon-α has been reported to inhibit the growth of meningioma cells in vitro. [154] The outcomes of six patients with recurrent unresectable meningioma who received INF-α 2b for five days a week showed that one patient had minor reduction of the tumor and four patients showed stable disease that lasted up to 14 months [155]. A longer and larger study on 12 patients with recurrent meningioma reported 9 patients who had stable disease after treatment with INF-α that lasted up to eight years [156]. A more recent study published on 35 patients with grade I recurrent meningioma, which received daily INF-α subcutaneously [157]. Ten patients had mild toxicity that required reduction of the drug dose, but overall the drug was safe. Twenty-five patients (74%) had stable disease with median time to tumor progression of seven months, and nine patients (26%) had progressed. Median survival time was eight months, and no radiographic response was found. At this stage, evidence that supports the use of interferons for meningioma is lacking [157]. Other immunotherapeutic approaches have not drawn much attention in the management of meningioma. Genetically defined meningioma models in immunocompetent mice such as the one triggered by *Nf*2 and *Cdkn2ab* inactivation [158], might help define and characterize the immune response induction and potential anti-meningioma efficacy after therapeutic interventions such as interferons and immune checkpoint inhibitors.

### 4.8. Oncolytic Virus

Oncolytic viruses (OV) are a class of biologic anti-tumor agents that selectively kill tumor cells leaving normal cells intact. [159] Different OVs have been investigated in clinical trials for different cancers, but there have been no meningioma OV trials. [159] In the past, a few publications explored OV therapy in preclinical meningioma models testing oncolytic adenovirus [160], and herpes simplex virus (oHSV) [1,161,162,163], although oncolytic adenovirus has not been studied in animal models. [162] Yazaki et al. showed that serum-cultured human malignant meningioma cell lines F5, GPSM4, and GPSM5 were permissive to oHSV G207 (γ34.5^−^, ICP6^−^). [162] Recently our group has established a patient-derived orthotopic malignant meningioma model (MN3) and investigated the therapeutic effect of G47Δ, an oHSV derived from G207, that lacks γ34.5 and α47 and has lacZ insertion inactivating ICP6 [1]. The MN3 model is *NF2* mutant, recapitulated the genotypic and phenotypic characteristics of clinical meningioma and serially reproduced orthotopic (subdural) tumors in mice. G47Δ was able to replicate in MN3 cells in vitro and single intratumoral injection of G47Δ extended overall survival of mice bearing intracranial MN3 tumors without any toxic effect. Moreover, G47Δ was able to replicate and kill several other human primary meningioma cultures in vitro [1]. The antitumor potency of G47Δ was shown in *NF2* mutant schwannoma models as well [164,165].

Currently, G47Δ is in clinical trials for recurrent glioblastoma in Japan, and preliminary results have suggested safety and encouraging efficacy. Our promising therapeutic effects of oHSV G47Δ in HGMs models both in vitro and in vivo support consideration of a clinical trial of G47Δ for refractory HGMs. Importantly, oncolytic viruses do not rely on specific mutations in tumors, rather common tumor features such as impaired innate immune response. The unique mechanism of action of oHSV enables killing of both *NF2* intact and mutant menigniomas and meningiomas that harbor other mutations. Furthermore, oHSV virotherapy elicits an anti-tumor cellular immune response, which is considered vital in anti-cancer efficacy. Preclinical meningioma models in immunocompetent animals might help advance studies of the anti-tumor immune system induced after virus infection, allowing designing more potent OVs or combination strategies with immune modulators with the goal to translate to the clinic to benefit patients.

## 5. Conclusions

The current standard of care for meningioma generally consists of surgery, followed by radiation therapy. Despite advances in surgery and radiotherapy, rates of recurrence are high in WHO grade II and III meningioma. To date, chemotherapy, hormonal therapy and immunotherapy have shown very limited benefit in meningioma patients. Multiple factors make basic science and clinical studies of meningioma challenging, including: The high tumor heterogeneity, the absence of accurate and reproducible preclinical platforms to test treatments and develop biomarkers, low disease prevalence (and therefore difficulty in recruiting patients with HGM), and finally, the lack of large prospective cohorts to perform conclusive clinical studies.

Progress in cancer genomics has provided us with vital molecular information about the genetic heterogeneity of meningiomas at an increasing rate, helping identify driver mutations, growth factors and their receptors, and critical signaling pathways. In this review we shined light on the latest and most promising studies of meningioma that have culminated in clinical trials. Those are examples of personalized medicine that goes after targets identified with next generation sequencing and pathways studies in the lab. The identification of genetic subtypes in meningioma has allowed a new clinical trial design that investigates different targeted agents assigned to patients stratified with tumor genotypes. Going forward trials need to be designed based on integrated diagnostic processes that include molecular and histologic diagnosis, thereby improving patient’s stratification and potentially response rates. The availability of preclinical xenograft and syngeneic animal models will help selecting targeted and non-targeted therapy. For instance, oncolytic virus can be combined with a variety of targeted treatments. OVs have a unique ability and efficacy against tumors with different genetic backgrounds. Therefore, having reliable preclinical platforms will help advance meningioma research and identification of promising treatments, both mono and combination therapies, accelerating translation to clinical testing.

The field holds promise in advancing novel therapies, targeted and non-targeted, identified by genome sequencing on patients and molecular pathogenesis studies in xenograft tumors. Such therapies can complement the current therapy and provide durable response. The incorporation of new advancements in basic science research and clinical practice has a potential to revolutionize meningioma treatment and improve patient’s quality of life. The neuro-oncology community is eager to see the clinical data of ongoing trials, summarized in Table 2, which has a potential to change the paradigm of meningioma management.

## Figures and Tables

**Table 1:** Genetic alterations in meningioma.

Gene	Mutation Type	Frequency (%)	Histopathological Subtype	Tumor Anatomical Location
*NF2*	Chromosome loss Var. mutations	40–60	Fibroblastic, transitional, atypical and anaplastic	Convexity and skull base
*TRAF7*	Var. mutations (WD40 domains)	12–25	Secretory, meningothelial and atypical	Skull base
*KLF4*	K409Q	9–12	Secretory	Skull base
*AKT1*	E17K	7–9	Meningothelial, transitional and atypical	Skull base
*TERT* promoter	C228T, C250T	6	Anaplastic and atypical (secondary)	Convexity and skull base
*POLR2A*	Q403K, L438_H439del	6	Meningothelial	Skull base (Tuberculum sellae)
*SMO*	L412F, W535L	1–5	Meningothelial and atypical	Anterior skull base
*PIK3CA*	H1047R most frequent	3–4	Meningothelial and transitional	Skull base
*SMARCE1*	Var. mutations	3–4	Clear cell	Spine and posterior fossa
*BAP1*	Var. mutations	Rare	Rhabdoid	Convexity and skull base

Var., various.

**Table 2:** Current ongoing clinical trials for patients with primary and recurrent meningiomas.

Targeted Pathway	Agent	Phase	Dates of the Study	Trial Identifier
SMO	Vismodegib	II	August 2015–present	NCT02523014
FAK	GSK2256098	II	August 2015–present	NCT02523014
mTORC1/2	AZD2014	II	August 2016–present	NCT02831257
mTOR	Everolimus	I	June 2013–present	NCT01880749
Somatostatin + radionucleotide	90-YDOTA tyr3-Octreotide	II	September 2017–present	NCT03273712
PD-1	Nivolumab	II	March 2016–present	NCT02648997
PDL-1	Pembrolizumab	II	November 2017–present	NCT03279692
PDL-1 + proton radiation	Avelumab	II	January 2018–present	NCT03267836
MEK1/2	Selumetinib	II	March 2017–present	NCT03095248
Histone Deacetylase	AR-42	I	September 2017–present	NCT02282917
CDK4/6	Ribociclib	I	October 2016–present	NCT02933736

Source: ClinicalTrials.gov.
